# Disordered eating in a Swedish community sample of adolescent girls: subgroups, stability, and associations with body esteem, deliberate self-harm and other difficulties

**DOI:** 10.1186/s40337-018-0189-z

**Published:** 2018-03-22

**Authors:** Njördur Viborg, Margit Wångby-Lundh, Lars-Gunnar Lundh, Ulf Wallin, Per Johnsson

**Affiliations:** 1 Department of Psychology, Lund University, Box 213, 221 00 Lund, Sweden; 2 Skånevård Sund, Child and Adolescent Psychiatry, Eating Disorders Centre Lund, Sweden, Baravägen 1, 221 85 Lund, Sweden

**Keywords:** Disordered eating, Risk factors, Adolescents, Cluster analysis, Prevention, Longitudinal, Cluster analysis

## Abstract

**Background:**

The developmental study of subtypes of disordered eating (DE) during adolescence may be relevant to understand the development of eating disorders. The purpose of the present study was to identify subgroups with different profiles of DE in a community sample of adolescent girls aged 13–15 years, and to study the stability of these profiles and subgroups over a one-year interval in order to find patterns that may need to be addressed in further research and prevention.

**Methods:**

Cluster analysis according to the LICUR procedure was performed on five aspects of DE, and the structural and individual stability of these clusters was analysed. The clusters were compared with regard to BMI, body esteem, deliberate self-harm, and other kinds of psychological difficulties.

**Results:**

The analysis revealed six clusters (Multiple eating problems including purging, Multiple eating problems without purging, Social eating problems, Weight concerns, Fear of not being able to stop eating, and No eating problems) all of which had structurally stable profiles and five of which showed stability at the individual level. The more pronounced DE clusters (Multiple eating problems including/without purging) were consistently associated with higher levels of psychological difficulties and lower levels of body esteem. Furthermore, girls that reported purging reported engaging in self-harm to a larger extent.

**Conclusions:**

Subgroups of 13–15 year old girls show stable patterns of disordered eating that are associated with higher rates of psychological impairment and lower body esteem. The subgroup of girls who engage in purging also engage in more deliberate self-harm.

## Plain English summary

In this study, we searched for different patterns of disordered eating among Swedish school girls, aged 13–15 years. We found five different patterns of disordered eating that were called Multiple eating problems including purging, Multiple eating problems without purging, Social eating problems, Weight concerns and Fear of not being able to stop eating. We also found a pattern free from disordered eating, called No eating problems. In general, these patterns remained relatively stable over a one-year period. The more severe disordered eating patterns (Multiple eating problems including/without purging) were consistently associated with higher levels of psychological difficulties and lower levels of body esteem. Furthermore, girls who reported purging also reported engaging in deliberate self-harm to a larger extent. In conclusion, there are subgroups of girls at this age for whom patterns of disordered eating are already established and associated with higher rates of psychological impairment, lower body esteem, and (when purging is involved) with higher rates of deliberate self-harm.

## Background

Disordered eating is a common problem. Although the lifetime prevalence of clinical eating disorders (ED) is limited to around 5% [1], studies show that subclinical forms of disordered eating (DE) are much more common, especially among adolescent girls (e.g., [2–12]).

Although the term disordered eating (DE) is sometimes confined to non-normative eating patterns which do not fulfil the criteria of a clinical ED [9], in the present study we use this term in a wider sense to include all individuals who suffer from some eating disorder symptoms, whether at a clinical or non-clinical level. Longitudinal studies indicate that DE remains stable or increases from adolescence to young adulthood (e.g., [13–15]). Studies have also demonstrated associations between DE and other psychological problems and lower levels of quality of life [16, 17].

An important question is how the various forms of DE should be classified. The development of increasingly sophisticated empirical methods (e.g., hierarchical cluster analysis and latent profile analysis) for the classification of individuals on the basis of their profiles of various characteristics makes it possible to carry out empirical studies of subgroups of individuals with different profiles of DE that have the potential to increase our understanding considerably. One advantage of this approach is that it can cover all kinds of DE, both at a clinical and a non-clinical level, and help to identify developments that occur over time, for example from less severe to more severe forms – which may be important knowledge for prevention purposes.

The purpose of the present study was to empirically identify different subgroups of girls with differing profiles of DE at the age of 13–15 years, and to study the stability of these profiles and subgroups over a one-year interval to see if this suggests any patterns that may need to be addressed in further research and prevention.

We are only aware of one cluster-analytic study of profiles of DE in community samples. Hansson et al. [3] analysed the patterning of four indicators (eating concern, restraint, shape concern, and weight concern) of DE as measured by the Eating Disorders Examination Questionnaire (EDE-Q) to identify typical DE patterns in a community sample of Swedish adolescents aged 13.5–19 years. The analysis resulted in a six-cluster solution for each gender. For girls, the two clusters that scored above the clinical cut-off on all DE indicators were associated with higher levels of depression, emotion dysregulation and lower levels of self-esteem. The profiles of these two clusters differed primarily by one of them showing particularly high scores on restraint.

Pattern analysis has also been applied to clinical samples of adult women with EDs. For example, Clinton, Button, Norring and Palmer [18] carried out separate cluster analyses of two samples of adult female patients from Sweden and England. Classifying the patients on the basis of 10 key clinical variables of diagnostic relevance (BMI, fear of weight gain, restriction of food intake, avoidance of fattening foods, binge eating, self-induced vomiting, abuse of laxatives, compulsive exercise, amenorrhea, and body-image disturbance), they arrived at a three-cluster solution in both samples (although they also reported their 4- and 5-cluster solutions in their article). The largest cluster, comprising 37.9% of the women in the Swedish sample and 45.3% of the English sample was called “Generalized Eating Disorder” and was characterized by high levels of pathology on all variables except weight and menstrual functioning. The second largest cluster was called “Anorexics” and was characterized by low weight, amenorrhea and the absence of binge eating. Finally, the third and smallest cluster was called “Overeaters” and was characterized by high weight and moderate levels of binge eating and compensatory behaviours. As the authors point out, although these patterns resemble existing diagnostic categories, they were far from identical with these. Of the patients who had a clinical diagnosis of AN, around three quarters fell into the “anorexics” cluster, and one quarter into the “generalized eating disorder” cluster. Of those who had a clinical diagnosis of BN, 61% fell into the “generalized eating disorder” cluster, whereas almost all of the others fell in the “overeaters” cluster. Of those who had a clinical diagnosis of BED, 94% were located in the “overeaters” cluster.

Although it is difficult to generalize from these results with clinical samples of adult female patients to a community sample of teenage girls, it is interesting to note that the largest cluster in Clinton et al.’s [18] study showed a pattern of “generalized eating disorder”, which combined both anorexic and bulimic symptoms. This suggests the hypothesis that we may find a cluster of generally disordered eating also among young adolescents – which is also consistent with the results from Hansson et al.’s [3] study. On the other hand, the three-cluster solution chosen by Clinton et al. [18] may be questioned, as they do not report any data on the heterogeneity of the clusters that were arrived at.

On the basis of these previous studies, we expected a Swedish community sample of 13–15 year old girls to contain one subgroup that could be characterized as “generally disordered eating”, with elevated levels of all symptoms of DE. In addition, on the basis of current classifications of eating disorders, we expected to find subgroups characterized by anorexic-like profiles, bulimic-like profiles, and BED-like profiles. Furthermore, we expected to find a “no eating problems profile”.

Because we used data from a 2-wave longitudinal study of a community cohort, we were able to replicate the same analyses with a one-year interval, to increase the validity of the conclusions. The longitudinal design also made it possible to study structural stability (i.e., whether similar patterns emerge at both measure points) and individual stability (i.e., whether adolescents who show one pattern at Time 1 tend to show a similar pattern at Time 2), and also to explore possible developmental trends in terms of individuals with one type of DE profile tending to change into another pattern during this one-year period. Finally, we also set out to compare these subgroups in terms of BMI, body esteem, self-harm, and other psychological difficulties.

## Methods

### Participants

The participants consisted of a community sample of all female students in two grades of regular school in a Swedish municipality who took part in a two-wave longitudinal study with a one-year interval. This municipality had approximately 40.000 inhabitants and was fairly representative of Sweden in terms of demographics, although slightly more rural, and with a slightly lower mean income level and lower educational level than the rest of Sweden (for more detailed information, see [19]). At Time 1, it contained five schools with 504 female students in Grades 7–8. At Time 2 there were 500 girls who attended Grades 8–9. The test–retest interval for the different schools ranged from 12 months and 7 days to 13 months and 11 days.

### Instruments

#### Disordered eating

Risk Behaviour Related to Eating Disorders (RiBED-8; [20]) is an eight-item instrument where participants are asked to rate statements about eating-related behaviours and attitudes as to how often each statement applies to them, on a scale from 1 to 4 (with the response alternatives “never”, “seldom”, “often”, and “very often”). The RiBED-8 was specifically designed to capture risk behaviour for eating disorders and has been found to be successful in predicting clinical eating disorders in a Danish sample [20]. The original Danish version of the RiBED-8 showed good psychometric properties for girls but not for boys. Also, the Swedish version of RiBED-8 (validated by Viborg et al. [21]) showed good test-retest reliability and construct validity for girls, but not for boys. In the present study, three RiBED-8 items (“I have a bad conscience because I eat sweets”, “I’m satisfied with my eating habits”, “I feel I have to control my eating either by maintaining a strict diet or in some other way”) were omitted from the cluster analyses, due to low test-retest reliability or unclear clinical relevance for the purposes of the present study. The RiBED-8 items that were used in the present study comprise the following questions: (1)“I diet”, (2)“I throw up to get rid of food that I have eaten”, (3)“I'm afraid of not being able to stop eating once I've started”, (4)“I feel that my desire to lose weight has completely taken over”, and (5)“I feel uncomfortable when I eat with others”. There were complete data on the five relevant items for 490 girls at T1 (i.e., representing 97.2% of all girls), and for 483 girls at T2 (i.e., representing 96.6% of all girls).

#### Psychological difficulties

The Strengths and Difficulties Questionnaire — self-report version (SDQ-s; [22]) is a widely used screening instrument for psychological problems among children and adolescents, which contains 25 statements. The participants are instructed to respond to each item on the basis of how things have been for them during the last six months. The items are divided into four difficulty scales (hyperactivity–inattention, emotional symptoms, conduct problems, peer problems) and one prosocial scale (with five items each). The items are scored 0 for “not true”, 1 for “somewhat true” and 2 for “certainly true”. The four first-mentioned scales are summed to generate a Total Difficulties score. The SDQ was translated into Swedish by Smedje et al. [23], and the self-report version was empirically validated by Lundh et al. [19]. According to their results, the psychometric properties of SDQ-s resembled findings from other language versions, in terms of acceptable test-retest stability but with low internal consistencies for some of the subscales, and evidence of good convergent and discriminant validity. In the present sample Chronbach’s alpha for the subscales was 0.67 for emotional symptoms, 0.66 for hyperactivity, 0.58 for conduct disorder, 0,59 for peer problems, and 0.77 for SDQ-Total Difficulties.

#### Body esteem

Body-Esteem Scale for Adolescents and Adults — Appearance subscale (BEAA; [24]) is a 10-item measure which assesses participants’ general satisfaction with their own appearance. Participants were asked to rate their degree of agreement with different statements (e.g., “I look as good as I'd like”, “My appearance bothers me” [reversed]). The instrument was translated into Swedish and validated by Erling et al. [25]. Whereas the original version uses a 5-point Likert scale, we used a 4-point Likert scale (so that the participants could use the same response format as with the RiBED-8). Lundh et al. [19] reported good internal consistency for this version of BEAA (alpha = .89). The internal consistency was also good for this sample (alpha = .92).

#### Deliberate self-harm

Deliberate self-harm was measured by a shortened and modified version of the Deliberate Self-Harm Inventory (DSHI) validated by Gratz [26] and adapted to adolescents by Bjärehed et al. [27]. In the present revised 9-item version of the inventory (called the DSHI-9r; [27]), respondents are asked if they have deliberately engaged in any of nine different forms of deliberate self-harm during the past 6 months. These nine forms were: cutting wrists, arms, or body areas; minor cutting causing bleeding; carving words, pictures, etc. into the skin; burning oneself with cigarette, lighter or match; severe scratching, causing bleeding; sticking sharp objects into the skin; biting oneself so that the skin is broken; punching oneself or banging one’s head, thereby causing a bruise; and preventing wounds from healing. (In the Swedish language, there is a differentiation between “skära” [which is deeper] and “rispa” [which is more superficial]. In the present study, this distinction has been translated into “cutting” and “minor cutting”). Respondents are instructed to rate the number of times, between 0 and 5 times, they have engaged in each of these behaviours, or if they have engaged in them “more than five times” (scored as 6). A total score was computed by summarizing the scores on all DSHI-9r items (ranging from 0 to 54). Lundh et al. [28] reported that the DSHI-9r showed good internal consistency (alpha = .90); evidence of good test-retest reliability for the earlier version DSHI-9 was reported by Bjärehed et al. [27]. The internal consistency was good for the present sample (alpha = 0.91).

#### Body mass index (BMI)

Body mass index was computed to compare the clusters regarding BMI. The questionnaire included one question about the participant’s length (with ten response alternatives, from “not taller than 150 cm”, and “151–155 cm” up to “186–190 cm”) and one question about weight (with eleven response alternatives, from “not more than 35 kg”, and “36–40 kg”, up to “76–80” and “more than 80 kg”). The midpoints of the weight and height intervals were used to compute BMI. Because BMI was based only on self-reports, and in terms of response categories, only approximate BMI is captured.

### Procedure

This research was conducted after approval by the Regional Ethics Committee in Lund. Contact was established with school managements via head-masters who gave consent to their schools’ participation in the study. Information about the form and purpose of the study was sent by mail to the parents, who were asked to contact the school teachers or the researchers if they did not want their child to participate. This passive consent procedure was considered the most ethically appropriate procedure considering the circumstances [29]. Parents as well as children were informed that this was a research project on the situation of young people today, in terms of how they feel, and how they perceive themselves, their feelings, relations, and life situation. The participants were also informed that their participation was voluntary, that they were free to withdraw at any time and without having to give a reason, that their answers were treated confidentially, and that no school personnel would have access to their answers. Contacts were established with representatives from school healthcare in the municipality to facilitate procedures if serious psychological problems or other circumstances related to participants would warrant an intervention.

The 11-page questionnaire was filled out in school, as part of a separate lecture hour, and was administered by research assistants from Lund University. A teacher was present, but did not participate in the data collection. In order to guarantee the students’ privacy, their school desks were separated as much as possible. The students were instructed to answer all questions as best they could, but not to think too much about any answer. They were instructed not to write their names anywhere on the questionnaire. After completion of the questionnaire, it was sealed in an envelope by the student.

### Statistical analysis

Cluster analysis was used to group all participants on the basis of their profiles of scores on five items from the RiBED-8. The cluster analysis was done in four steps in accordance with the LICUR procedure [30]. First, multivariate outliers were identified by means of a residue procedure and removed from further analysis. Second, Ward’s hierarchical clustering method was applied. Four criteria presented by Bergman [31] were used to decide on the optimal cluster solution: (a) theoretical meaningfulness of the cluster solution; (b) a cluster solution with k clusters is preferable to one with k-1 if a sharp decrease in explained error sum of squares (EESS) occurs between the solution with k clusters and with k-1 clusters; (c) the number of clusters should not be more than 15 and should not be expected to be less than five; (d) the size of the EESS for the chosen cluster solution should preferably not be less than 67% and at the very least exceed 50%. In addition, the homogeneity coefficient of each cluster should preferably be *<* 1. Third, a data simulation was undertaken to verify that the EESS was higher than what could be expected on a random data set with the same general properties as the data set used in the real analysis. Fourth, a non-hierarchical relocation procedure was carried out in order to improve the homogeneity of the clusters and to increase the variance explained by the cluster solution. The statistical package for pattern-oriented analyses ROPstat [30] was used for the main analyses. In addition, the CENTROID module in SLEIPNER [32] was used to study the structural stability of the cluster solutions by comparing the centroids of values on the five RiBED-8 items between T1 and T2. Finally, the cluster solutions from T1 and T2 were cross-tabulated to investigate individual stability and change over the studied one-year period. This was done by exact tests on single cells in two-way contingency tables using hypergeometric probabilities. The hypotheses of individual stability for each cluster were tested without correction for mass significance, whereas the exploration of streams between dissimilar clusters from T1 to T2 was tested using Bonferroni correction.

## Results

### Cluster analyses

At T1, 13 multivariate outliers were identified by means of the residue procedure and removed from further analysis, thus leaving 477 individuals for the cluster analysis. At T2, 5 multivariate outliers were identified by means of the residue procedure and removed from further analysis, thus leaving 478 individuals for the cluster analysis. Of these, there were full data on 422 girls at both T1 and T2.

#### Cluster solutions

**Fig. 1:**
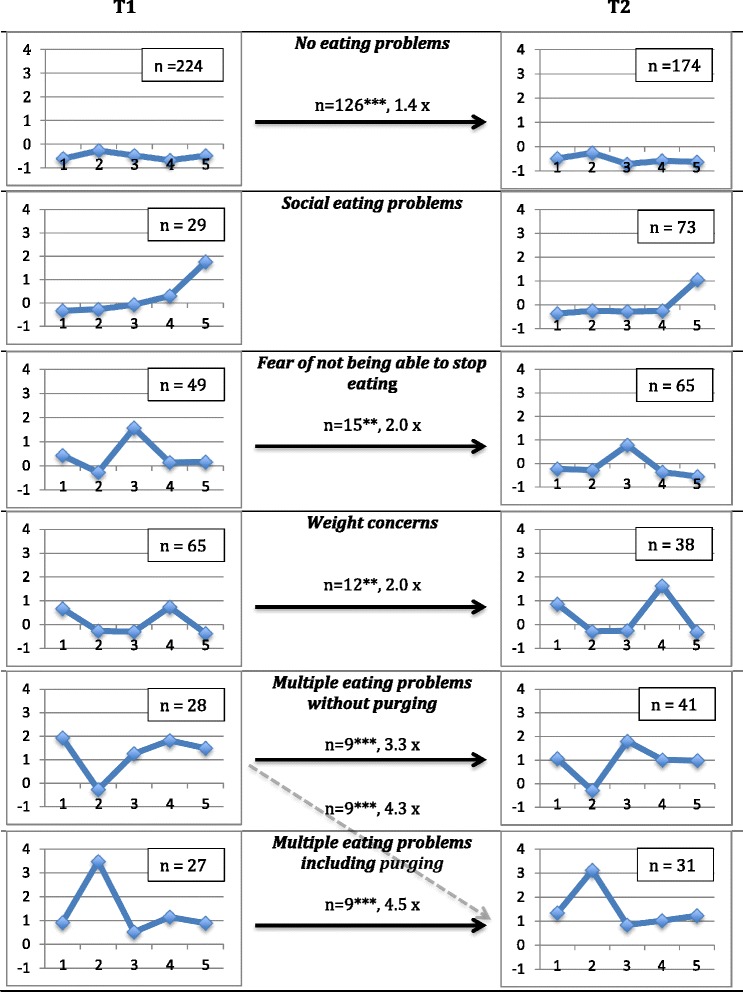
Cluster profiles at T1 and T2 in terms of z-scores. Full arrows indicate significant developmental (individual) stability. When no arrow is depicted it means that there was no individual stability. The figure also shows the strength of this stability; for example, there were 1.4 times as many girls as expected by chance who belonged to the No eating problems cluster at T1 and stayed in the same cluster at T2. The dotted arrow indicates a significant change. 1 = “I diet”; 2 = “I throw up to get rid of food that I have eaten”; 3 = “I’m afraid of not being able to stop eating once I’ve started”; 4 = “I feel that my desire to lose weight has completely taken over”; 5 = “I feel uncomfortable when I eat with others”. *p < .05; **p < .01; ***p < .001

In total, 477 cases were included in the cluster analysis at T1 that resulted in a six-cluster solution (see Table 1 for raw scores and Fig. 1 for Z-scores). Repeating this procedure at T2 with 478 cases also resulted in a six-cluster solution. The clusters were named in accordance with the peaks of their profiles. Data simulation showed that the explained ESS at both T1 (63.75% of the total ESS) and T2 (62.67%) was significantly higher than expected by chance (*p* < .01). The clusters were named: No eating problems (50.4% of the participants at T1, 40.2% at T2); Social eating problems (7.5% at T1, 16.6% at T2); Fear of not being able to stop eating (11.4% at T1, 14.9% at T2); Weight concerns (14.9%, 9.5% at T2); Multiple eating problems without purging (6.1% at T1, 9.9% at T2); and Multiple eating problems including purging, or for short the Purging cluster (6.9% at T1, 7.9% at T2).

**Table 1:** Description of the clusters in terms of number of participants, percentages, homogeneity coefficient (hc), and unstandardized mean values and standard deviations for the RiBED items at T1 (and at T2 in parentheses). Comparison on RiBED items by MANOVA

Clusters				RiBED-8 items									
	Number	Percent	hc	I diet		I throw up		I’m afraid of not being able to stop eating		Desire to lose weight that has taken over		Uncomfortable when I eat with others	
				M	SD	M	SD	M	SD	M	SD	M	SD
No eating problems	247 (194)	50.4 (40.2)	0.27 (0.25)	1.10^a^ (1.29^a^)	0.31 (0.52)	1.00^a^ (1.01^a^)	0.00 (0.10)	1.23^a^ (1.00^a^)	0.42 (0.00)	1.13^a^ (1.17^a^)	0.33 (0.38)	1.08^a^ (1.00^a^)	0.28 (0.00)
Social eating problems	37 (80)	7.5 (16.6)	0.84 (0.72)	1.32^a^ (1.40^a^)	0.53 (0.54)	1.00^a^ (1.01^a^)	0.00 (0.11)	1.57^b^ (1.39^b^)	0.50 (0.49)	2.00^b^ (1.48^a^)	0.67 (0.57)	2.81^d^ (2.29^b^)	0.66 (0.58)
Fear of not being able to stop eating	56 (72)	11.4 (14.9)	1.03 (0.49)	1.93^b^ (1.51^a^)	0.76 (0.56)	1.00^a^ (1.00^a^)	0.00 (0.00)	2.93^d^ (2.32^c^)	0.63 (0.55)	1.86^b^ (1.38^a^)	0.70 (0.49)	1.59^b^ (1.06^a^)	0.53 (0.23)
Weight concerns	73 (46)	14.9 (9.5)	0.58 (0.93)	2.12^b,c^ (2.41^b^)	0.47 (0.86)	1.00^a^ (1.00^a^)	0.00 (0.00)	1.38^a,b^ (1.41^b^)	0.49 (0.58)	2.40^c^ (3.22^c^)	0.64 (0.59)	1.18^a^ (1.24^a^)	0.38 (0.48)
Multiple eating problems without purging	30 (48)	6.1 (9.9)	1.60 (1.50)	3.13^d^ (2.58^b,c^)	0.57 (0.74)	1.00^a^ (1.00^a^)	0.00 (0.00)	2.67^d^ (3.21^d^)	0.99 (0.62)	3.37^e^ (2.65^b^)	0.67 (0.91)	2.60^d^ (2.23^b^)	0.93 (0.93)
Multiple eating problems including purging	34 (38)	6.9 (7.9)	2.57 (2.22)	2.32^c^ (2.82^c^)	1.04 (0.77)	2.09^b^ (2.29^b^)	0.29 (0.46)	2.06^c^ (2.37^c^)	0.92 (0.97)	2.76^d^ (2.66^b^)	0.92 (0.88)	2.15^c^ (2.42^b^)	0.92 (0.79)

Means with different superscripts differ significantly, according to post-hoc tests by Tukey HSD

#### Overview of the cluster solutions

A multivariate analysis of variance (MANOVA) was carried out to compare the clusters on their scores on the RiBED items. As seen in Table 1, the two multi-problem clusters differed significantly in most respects. At T1, the Purging cluster scored significantly higher on purging than the multi-problems cluster without purging, but at the same time it scored significantly *lower* on all other aspects of disordered eating. At T2, the differences were less pronounced but went in the same direction; although the Purging cluster scored higher on purging, it scored significantly lower on fear of not being able to stop eating.

The Weight concerns cluster scored significantly higher than the No eating problems cluster and the Social eating problems cluster on dieting and desire to lose weight (at both T1 and T2) and on fear of not being able to stop eating (at T2). Interestingly, at T2 they scored significantly higher than all other clusters (including both of the multi-problems clusters) on desire to lose weight that has taken over.

The cluster characterized by fear of not being able to stop eating scored consistently higher only on this specific item. Interestingly they scored significantly higher on this item at both T1 and T2 than three of the other clusters (No eating problems; Social eating problems, and Weight concerns), and at T1 they even scored higher than the Purging cluster on this item. The Social eating problems cluster scored consistently high on the item of feeling uncomfortable when eating with others, but they also scored higher than the No eating problems cluster on fear of not being able to stop eating.

#### Homogeneity of clusters

As seen in Table 1, the most homogeneous cluster was No eating problems (T1 hc = 0.27; T2 hc = 0.25; a low value means high homogeneity) and the most heterogeneous cluster was the Purging cluster (T1 hc = 2.57; T2 hc = 2.22).

#### Structural stability

Visual inspection of the profiles of the clusters in Fig. 1 suggests that the 6 clusters from T1 were fairly well replicated at T2. Analysis of the similarity of these profiles in terms of Average Squared Euclidian Distances (ASED) confirmed this for all clusters (ASED scores below .50 are assumed to indicate good similarity; L. R. Bergman, personal communication, December 18, 2017). These were, in decreasing order of similarity: No eating problems (ASED = 0.02), Multiple eating problems including purging (ASED = 0.11), Weight concerns (ASED = 0.17), Social eating problems (ASED = 0.17), Fear of not being able to stop eating (ASED = 0.37), and Multiple eating problems without purging (ASED = 0.39).

#### Individual stability and change from T1 to T2

A specific pattern is seen as individually stable if more individuals than can be expected by chance are observed to show this pattern at both T1 and T2. As seen in Fig. 1, five of the clusters showed significant individual stability: No eating problems (Observed: 126, Expected: 92.4, *p* < .001); Fear of not being able to stop eating (O: 15, E: 7.5, *p* < .01); Weight concerns (O: 12, E: 5.9, *p* < .01); Multiple eating problems without purging (O: 9, E: 2.7, *p* < .001); and Multiple eating problems including purging (O: 9, E: 2.0, *p* < .001). One cluster, the Social eating problems cluster did not show individual stability (O: 5, E: 5.0, *p* = 1.000). The occurrence of movements between dissimilar clusters from T1 and T2 was also explored. One significant movement was found (depicted by the dotted arrow in Fig. 1), from the Multiple eating problems without purging cluster at T1 to the Purging cluster at T2 (O = 9.0, E = 2.1, *p* < 0.05/30 = 0.002 after a Bonferroni correction).

#### Comparisons of the clusters on self-harm (DSHI-9r), body esteem (BEAA), psychological difficulties (SDQ), and BMI at T1

At T1 (see Table 2) the more severe eating problem clusters (Multiple eating problems without purging, Multiple eating problems including purging) had significantly higher mean values than all the other clusters on psychological difficulties (SDQ Total difficulties) and significantly lower scores than other clusters on body esteem (BEAA). With regard to self-harm (DSHI-9r) the Purging cluster stood out as scoring significantly higher than all the other clusters. As to BMI, both of the multi-problem clusters, the Weight concerns cluster, and the Fear of not being able to stop eating cluster scored significantly higher than the No eating problems cluster.

**Table 2:** Means (and standard deviations) on body mass index (BMI), self-harm (DSHI-9r), body esteem-appearance (BEAA), and SDQ measures of psychological difficulties, hyperactivity/ inattention, emotional symptoms, peer problems, and conduct problems for the clusters at T1, and comparison by one-way ANOVA

Cluster	BMI	DSHI-9r	BEAA	Total Difficulties	Hyperactivity/inattention	Emotional symptoms	Peer problems	Conduct problems
No eating problems	18.60^a^ (2.32)	1.78^a^ (5.55)	30.52^a^ (5.89)	8.27^a^ (4.24)	3.09^a^ (2.02)	2.55^a^ (1.97)	1.40^a^ (1.44)	1.26^a^ (1.29)
Social eating problems	19.76^a,b^ (2.55)	4.46^a,b^ (8.87)	28.03^a,b^ (5.80)	11.49^b^ (4.91)	4.43^b,c^ (1.88)	3.65^a,b^ (1.95)	1.69^a,b,c^ (1.58)	1.57^a^ (1.14)
Fear of not being able to stop eating	20.38^b^ (2.85)	4.29^a,b^ (5.93)	25.81^b^ (6.56)	11.34^b^ (4.61)	4.05^a,b,c^ (2.10)	3.45^a^ (2.06)	1.98^a,b,c^ (1.40)	1.86^a,b^ (1.24)
Weight concerns	20.63^b^ (3.09)	3.16^a,b^ (6.34)	24.80^b,c^ (6.42)	10.51^a,b^ (4.24)	3.96^a,b^ (2.16)	3.29^a^ (1.96)	1.46^a,b^ (1.37)	1.95^a,b^ (1.47)
Multiple eating problems without purging	20.82^b^ (2.58)	6.69^b^ (12.47)	20.13^d^ (5.20)	14.70^c^ (4.76)	5.23^c^ (2.13)	4.73^b,c^ (2.38)	2.27^b,c^ (1.31)	2.47^b^ (1.31)
Multiple eating problems including purging	20.24^b^ (2.52)	11.73^c^ (12.45)	21.88^d,c^ (7.70)	14.79^c^ (5.59)	4.47^b,c^ (2.12)	5.21^c^ (2.42)	2.53^c^ (1.86)	2.59^b^ (1.60)

Means with different superscripts differ significantly, according to post-hoc tests by Tukey HSD

#### Comparison between the clusters on self-harm (DSHI-9r), body esteem (BEAA), psychological difficulties (SDQ) and BMI at T2

At T2, a similar picture emerged (see Table 3). The Purging cluster had a significantly higher mean value on compared to all other clusters. This cluster also differed significantly from all other clusters except the Multiple eating problems without purging cluster on body esteem (BEAA) and on psychological difficulties (SDQ-Total Difficulties). As to BMI, the Multiple eating problems cluster without purging scored significantly higher than the No eating problems cluster and two other clusters (Social eating problems, and the Fear of not being able to stop eating), whereas the Purging cluster scored significantly higher than only the No eating problems cluster.

**Table 3:** Means (and standard deviations) on body mass index (BMI), self-harm (DSHI-9r), body esteem-appearance (BEAA), and SDQ measures of psychological difficulties, hyperactivity/ inattention, emotional symptoms, peer problems, and conduct problems for the clusters at T2, and comparison by one-way ANOVA

Cluster	BMI	DSHI-9r	BEAA	SDQ Total Difficulties	Hyperactivity/inattention	Emotional symptoms	Peer problems	Conduct problems
No eating problems	19.88.^a^ (2.74)	2.05^a^ (4.57)	29.96^a^ (6.21)	8.69^a^ (4.46)	3.23^a^(2.01)	2.65^a^ (1.96)	1.35^a,b^ (1.37)	1.47^a^ (1.43)
Social eating problems	20.25^a,b^ (2.60)	4.26^a^ (7.60)	25.55^b,c^ (6.42)	11.28^b,c^ (4.58)	4.04^a,b^ (2.00)	3.68^a,b^ (2.13)	1.91^a,b,c^ (1.44)	1.64^a^ (1.33)
Fear of not being able to stop eating	20.12^a,b^ (2.75)	2.91^a^ (4.80)	27.25^a,b^ (6.12)	10.23^a,b^ (3.90)	3.79^a,b^ (2.00)	3.52^a^ (2.11)	1.29^a^ (1.26)	1.64^a^ (1.44)
Weight concerns	21.31^a,b,c^ (3.02)	2.66^a^ (6.42)	24.25^b,c^ (5.94)	10.39^a,b^ (3.46)	4.03^a,b^ (1.58)	3.50^a^ (1.86)	1.24^a^ (1.20)	1.63^a^ (1.02)
Multiple eating problems without purging	21.82^c^ (2.91)	5.84^a^ (7.01)	22.52^c,d^ (5.49)	13.36^c,d^ (5.54)	4.43^b,c^ (2.07)	4.76^b,c^ (2.31)	2.17^b,c^ (2.01)	2.00^a,b^ (1.67)
Multiple eating problems including purging	21.52^b,c^ (2.50)	14.47^b^ (16.12)	20.26^d^ (6.21)	15.24^d^ (5.73)	5.18^c^ (2.22)	4.88^c^ (2.32)	2.47^c^ (1.99)	2.48^b^ (1.72)

Means with different superscripts differ significantly, according to post-hoc tests by Tukey HSD

## Discussion

To the knowledge of the authors, this is the first study to use a longitudinal design to identify specific patterns of disordered eating (DE) among a community sample of adolescent girls, studying the structural stability of profiles and individual stability of these subgroups over a one-year period. Altogether, 6 clusters were identified at T1 and at T2, all of which had structurally stable profiles and five of which also showed stability at the individual level over a one-year period. The results confirmed the hypothesis that we would find a cluster of girls characterized by “generally disordered eating”, with elevated levels on most aspects of DE. This was seen most clearly in the Purging cluster, which represented 7–8% of the girls. This cluster also represents the most problematic group in terms of potential psychopathology, as seen in its association with seelf-harm.

The next most problematic cluster was the Multiple eating problems without purging cluster, which constituted 6–10% of the girls. This cluster tended to have the highest scores (i.e., even higher than the Purging cluster) on all aspects of DE except purging. Interestingly, there was a significant progression among a subgroup of girls from the multi-problem DE pattern without purging to the one with purging. This suggests that there is an elevated risk of developing purging as an additional symptom for those who have a pattern of previously elevated levels on other symptoms of DE. The knowledge of such a pathway might inform prevention efforts.

Although we found no cluster with an anorexia-like profile, we found some support for a BED-like cluster in the Fear of not being able to stop eating cluster (11–15% of the girls). Two other DE clusters were also found: Social eating problems (7.5% at T1 and 16.6% at T2) and Weight concerns (14.9% at T1 and 9.5% at T2). The Social eating problems cluster was the only one that did not show individual stability (although it was structurally stable). Structural stability among the present clusters means that the same patterns of symptoms are likely to appear on a regular basis. Individual stability, on the other hand, means that the same individuals are likely to show the same patterns over time. The present results show that in general the identified patterns seem to be relatively stable over time, with the exception for the Social eating problems cluster. In other words, most of the DE clusters that were identified were not transient states but showed some stability over at least one year. Moreover, as seen in Fig. 1, the more severe clusters were the most stable (with fourfold risk increases of still having the same pattern one year later). However, the present results cannot tell if these patterns are stable beyond a one-year period, or to what extent these girls have clinical eating disorders, or are likely to develop clinical disorders. Further longitudinal studies are needed to answer that question. The stability of DE clusters is in line with previous reports on stable DE symptoms [13–15].

Several interesting findings were noticeable regarding the associations between the eating clusters and BMI, self-harm, psychological difficulties and body esteem. The more pronounced DE clusters (Multiple eating problems including/without purging) were consistently associated with higher levels of psychological difficulties as well as lower levels of body esteem. The results resemble the associations between DE clusters and more severe levels of psychological problems described in the Hansson et al. study [3]. The associations between DE and psychological symptoms such as depression have also been described by Herpertz-Dahlmann et al. [15].

Regarding BMI, Herpertz-Dahlmann et al. [14] showed in a longitudinal study of DE among 11–17 year-olds that having more DE symptoms at baseline was associated with higher levels of obesity at follow up 6 years later. A longitudinal study [8] on DE among a community sample of 7082 adolescents aged 13–15 years found three ED symptoms dimensions: bingeing/overeating, weight/shape concern and weight-control behaviours, and food restriction. Bingeing/overeating and weight/shape concern and weight-control behaviours predicted higher BMI at follow up two years later, and restrictive eating predicted lower BMI. The present study did not show any clear differentiation of this kind, all DE clusters tending to be associated with higher BMI than the No eating problems cluster; it is possible, however, that this is due to the absence of a longer follow-up period. Also, the fact that BMI was not measured precisely, but based on response categories which only allowed for an approximate BMI, may have contributed to other results than studies where it was captured exactly.

An interesting finding is that only the Purging cluster was consistently associated with substantially and significantly higher levels of self-harm – that is, girls who reported purging also reported engaging in self-harm to a greater extent. This finding was present at both T1 and T2. The present results are in line with reviews of previous research which has suggested that deliberate self-harm is frequent in EDs, and especially in EDs that include bingeing and purging behaviors [33], and that self-harm is more frequent in people with bulimia than in anorexia [34].

One cluster, the Social eating problems cluster, was not stable at an individual level from T1 to T2, although it was a structurally stable phenomenon (between T1 and T2). This cluster may represent a temporary phase that occurs regularly in a certain percentage of adolescents (e.g., as an expression of increasing self-consciousness), but at an earlier age in some adolescents (e.g., at T1) than in in others (who are in this developmental phase at T2). It may be questioned whether the Social eating problems cluster should be considered a DE cluster; maybe it represents a form of social anxiety rather than DE? What still speaks somewhat in favour of some form of DE being involved, however, is that the individuals in this cluster scored consistently higher than the No eating problems cluster on fear of not being able to stop eating.

### Strengths and limitations

The present study has several strengths. It is possibly the first longitudinal study to investigate patterns of DE in a community sample of adolescent girls by means of an advanced procedure for cluster analysis. The longitudinal design with high response rates at both T1 and T2 adds to the validity of the results. Furthermore, the use of several different instruments to measure different aspects of psychological problems (psychological difficulties, self-harm and body dissatisfaction) gives a broad picture of the associations between DE clusters and other forms of psychological problems/psychopathology.

In addition, we used an advanced form of cluster analysis, with explicit analyses of explained error sum of squares (EESS), homogeneity coefficients, and significance testing of the cluster solutions. This showed that the cluster solutions were statistically significant and explained a reasonable amount of variance. The homogeneity coefficients were consistently less satisfactory for the two multi-problems clusters, but this is not unexpected because these clusters could be characterized as “extreme clusters,” whose lower homogeneity is the result of having more extreme cases. Whereas there is only one way of having no problems (as exemplified in the homogeneity of the No eating problems cluster) there are several ways of having high scores on problem items (as seen in the heterogeneity of the two multi-problem clusters).

There are several limitations of the present study. First, it is based entirely on self-assessment instruments. In addition, the RiBED-8 inventory uses the scaling “never”, “seldom”, “often” and “very often” which may have been interpreted differently by different respondents; for example, some respondents may have interpreted “often” as “once a week” while others may have interpreted it as “daily”. Furthermore, a more multifaceted approach with structured interviews, and peer, parent or teacher reports could have generated stronger conclusions. Second, the sample came from only one Swedish municipality and although this municipality was relatively representative of Sweden in general, there is always the possibility of a bias in a sample based on a certain geographic location. It may therefore be of interest to replicate this kind of study in other contexts. Third, there was no follow-up after the one-year period that could have confirmed potential ED diagnoses, and consequently we do not know to what extent the present results are generalizable to adolescent girls with clinical eating disorders. Future research could be aimed at further follow-up of the present DE clusters in terms of the eventual development of clinical EDs. Fourth, due to lacking validity for the RiBED-8 inventory for boys [21] they were not included in the present analyses. Fifth, BMI was measured only by self-report, and in terms of different response categories, which means that only approximate BMI is captured.

Finally, the decision to conduct the cluster analysis only on RiBED items, and not to include BMI and/or body esteem as variables in the cluster analysis, may be questioned. This was a choice made on the assumption that a cluster analysis should be based on variables that belong to the same “system” [35], and that the system in focus here was disordered eating. Although body dissatisfaction (body esteem) is known to be empirically related to DE, we considered it an associated variable, rather than an aspect of DE. Similar considerations apply to the BMI variable – although BMI is empirically associated with DE  eating it is not an aspect of DE. As pointed out by Bergman [35], there are "different levels of analysis. What is a whole system at one level might just be a component at a higher level, and in any given study, only one or a few levels can be considered" (p. 30). The choice of DE as the focused level of analysis in the present study, therefore, is in a certain sense arbitrary; we might as well have chosen to focus on a system at a higher level that includes BMI and body esteem as well as aspects of DE. Although this might have made the cluster solutions more comparable to clinical diagnoses of eating disorders, however, our focus was on DE as a phenomenon in itself.

## Conclusions

In conclusion, the present study found that subgroups of 13–15 year old girls show stable patterns of disordered eating that are associated with higher rates of psychological impairment and lower body esteem and, furthermore, that the subgroup of girls who engage in purging also engage in more deliberate self-harm.
